# Gastrointestinal Stromal Tumor (GIST) in a Young Adult: A Rare Presentation and a Multidisciplinary Approach

**DOI:** 10.7759/cureus.68001

**Published:** 2024-08-28

**Authors:** Bibek Karki, Mahpara Munir, Abinash Parajuli, Sisira Santhosh, Adiraj Singh

**Affiliations:** 1 Internal Medicine, Hurley Medical Center - Michigan State University, Flint, USA; 2 Internal Medicine and Pediatrics, Hurley Medical Center - Michigan State University, Flint, USA

**Keywords:** rare cancer, high-grade stromal tumor, constipation, abdominal pain, gist

## Abstract

A gastrointestinal stromal tumor is a rare gastrointestinal tumor of mesenchymal origin. We present a rare case of a 32-year-old male patient with a history of iron deficiency anemia who presented with nocturnal cramping abdominal pain, nausea, non-bloody vomiting, loss of appetite, and weight loss. The patient had no significant family history of cancer. Prior imaging showed gastric distension with chronic inflammatory changes, but a scheduled esophagogastroduodenoscopy (EGD) was not done due to loss of follow-up. On admission, the patient was tachycardic with anemia. An abdominal CT scan showed new areas of gastric wall thickening with edematous wall thickening at the gastric cardia. An EGD revealed deep gastric ulcers with elevated edges with central necrosis, raising concerns for malignancy. A biopsy of the gastric cardia confirmed a high-grade stromal tumor with the aid of a DOG1 test; the gastric cardia was KIT-positive in immunohistochemical testing but negative for Helicobacter pylori. The tumor was staged at pT3N0. He was treated with surgical resection with negative margins and imatinib therapy. Postoperative surveillance showed no evidence of malignancy and the patient experienced a positive response to treatment with stable hemoglobin levels and significant weight gain.

## Introduction

Gastrointestinal stromal tumors (GISTs) are rare slow-growing gastrointestinal (GI) tract tumors arising from primitive mesenchymal cells, usually located in the stomach (60%) and small intestine (25%) [1]. The age-adjusted yearly incidence rate of malignant GISTs is 0.68 in 100,000, with higher rates among men and blacks [1]. The average age of presentation of these tumors is 60-65 years, and they are even rarer among young adults [2]. 

GISTs are believed to have originated from Cajal interstitial cells, mesenchymal cells found in the muscle layers of the alimentary canal [3]. They arise from the mutations of KIT proto-oncogene, or PDGFRA (PDGF receptor alpha) genes [4,5]. However, KIT/PDGFRA wild-type GIST does not harbor mutations in KIT/PDGFRA genes; instead, it is a result of a mutation in succinate dehydrogenase, NF1, BRAF, and KRAS [6].

The exact cause of GIST is unknown in most cases, but a small portion of the cases are related to genetic factors [3]. The usual presentation is abdominal discomfort, abdominal fullness, and upper GI bleeding, and it is usually treated with surgery and chemotherapy agents like imatinib. We present a case of a relatively young patient diagnosed with GIST, which prompted further investigation and raised suspicion for an underlying genetic mutation or syndrome.

## Case presentation

A 32-year-old male with a history of iron deficiency anemia presented to the emergency department with nocturnal cramping abdominal pain, nausea, and bloody vomiting for two weeks. They were associated with abdominal bloating, loss of appetite, weight loss, constipation without obstipation, and weakness. He denied a history of eating disorders. There was no family history of known GI malignancy or autoimmune disease. A prior computed tomography (CT) scan showed gastric distension with chronic inflammatory changes in the antrum and the first and second part of the duodenum two months ago. An esophagogastroduodenoscopy (EGD) was scheduled in the outpatient department two months ago but was not done.

The patient was alert, afebrile, and normotensive, but tachycardic; the tachycardia improved with hydration and pain control. The patient had anemia with hemoglobin at 6.0 and mild leukocytosis at 13.1 but had a normal erythrocyte sedimentation rate (ESR) and C-reactive protein (CRP). Hemoglobin improved to 8.1 after two units of blood transfusion. The liver function test (LFT), thyroid function test (TSH), lipase, vitamin B12, and folate levels were normal. The workup for celiac disease with anti-endomysial antibody and anti-tissue transglutaminase antibody was negative. The HIV test was negative. 

**Figure 1:**
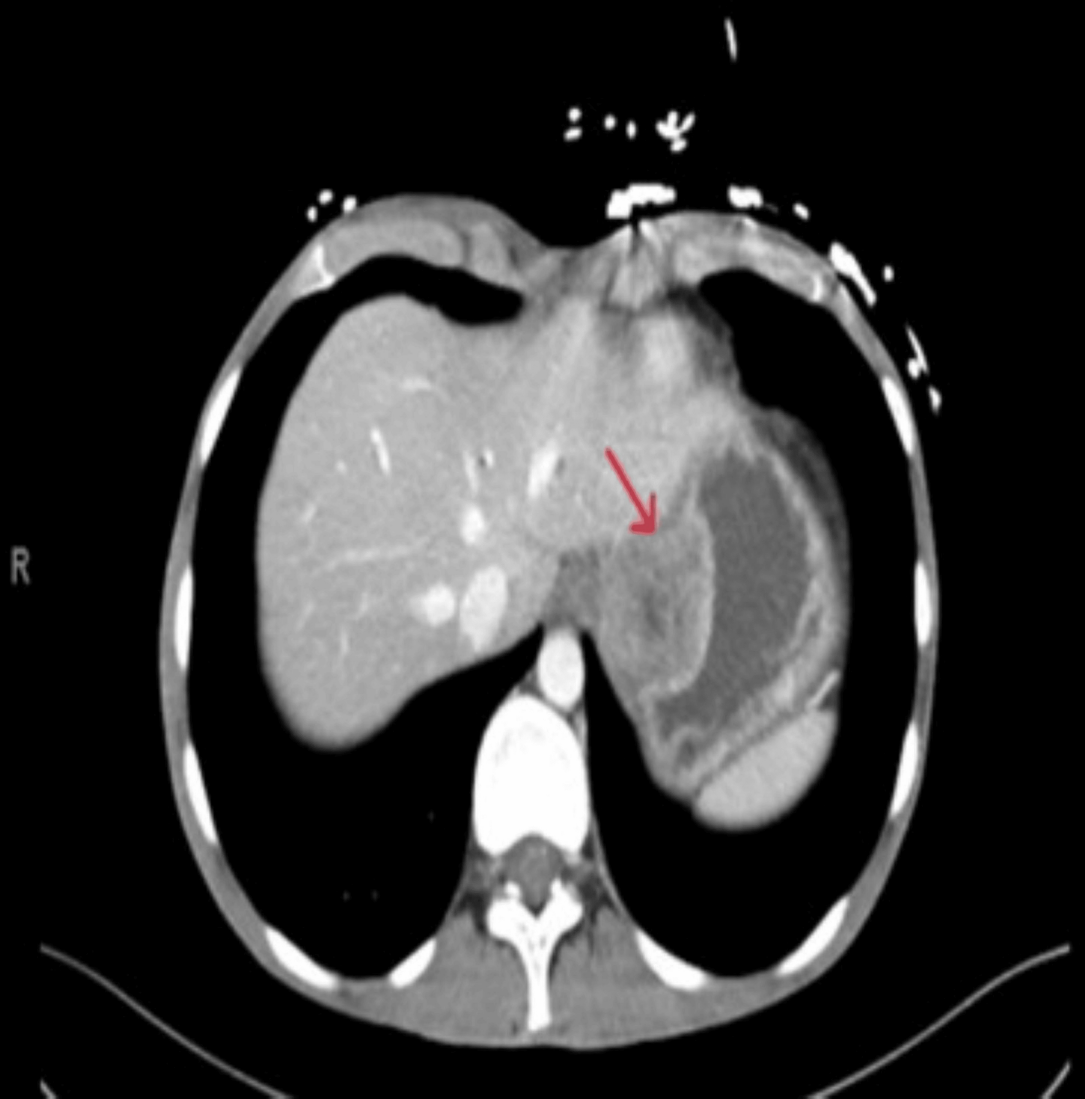
CT of the abdomen revealing a mass in gastric cardia (red arrow)

A CT scan of the abdomen and pelvis showed gastric distension with areas of edematous wall thickening at the level of gastric cardia, as shown in Figure 1.

**Figure 2:**
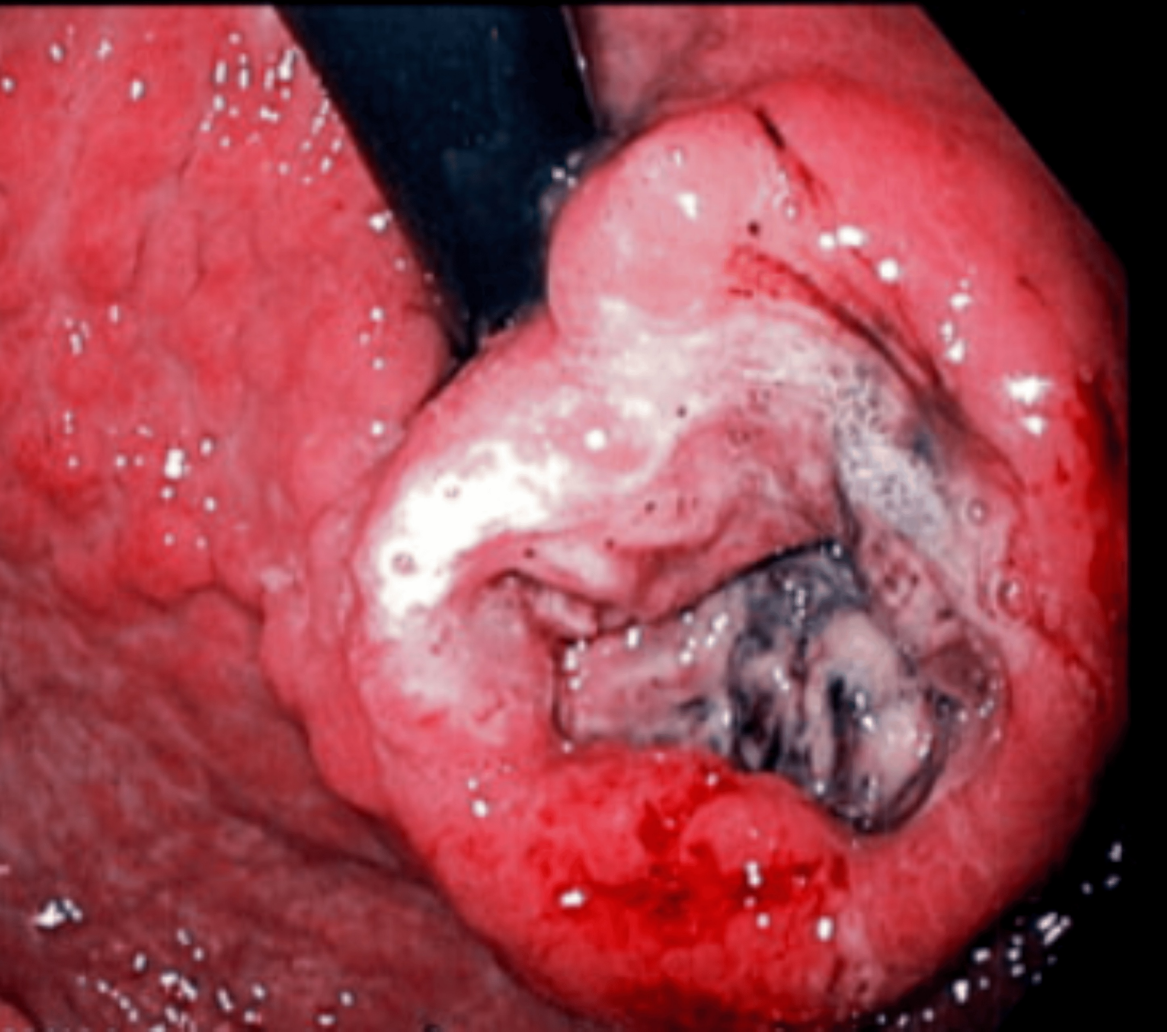
Esophagogastroduodenoscopy showing deep ulcer in the cardia with elevated edges and central necrosis, raising concerns of malignancy

An EGD showed esophagitis, gastritis with deep ulceration, and central necrosis at the gastric cardia, raising concerns of malignancy, as shown in Figure 2. Multiple biopsies were taken.

**Figure 3:**
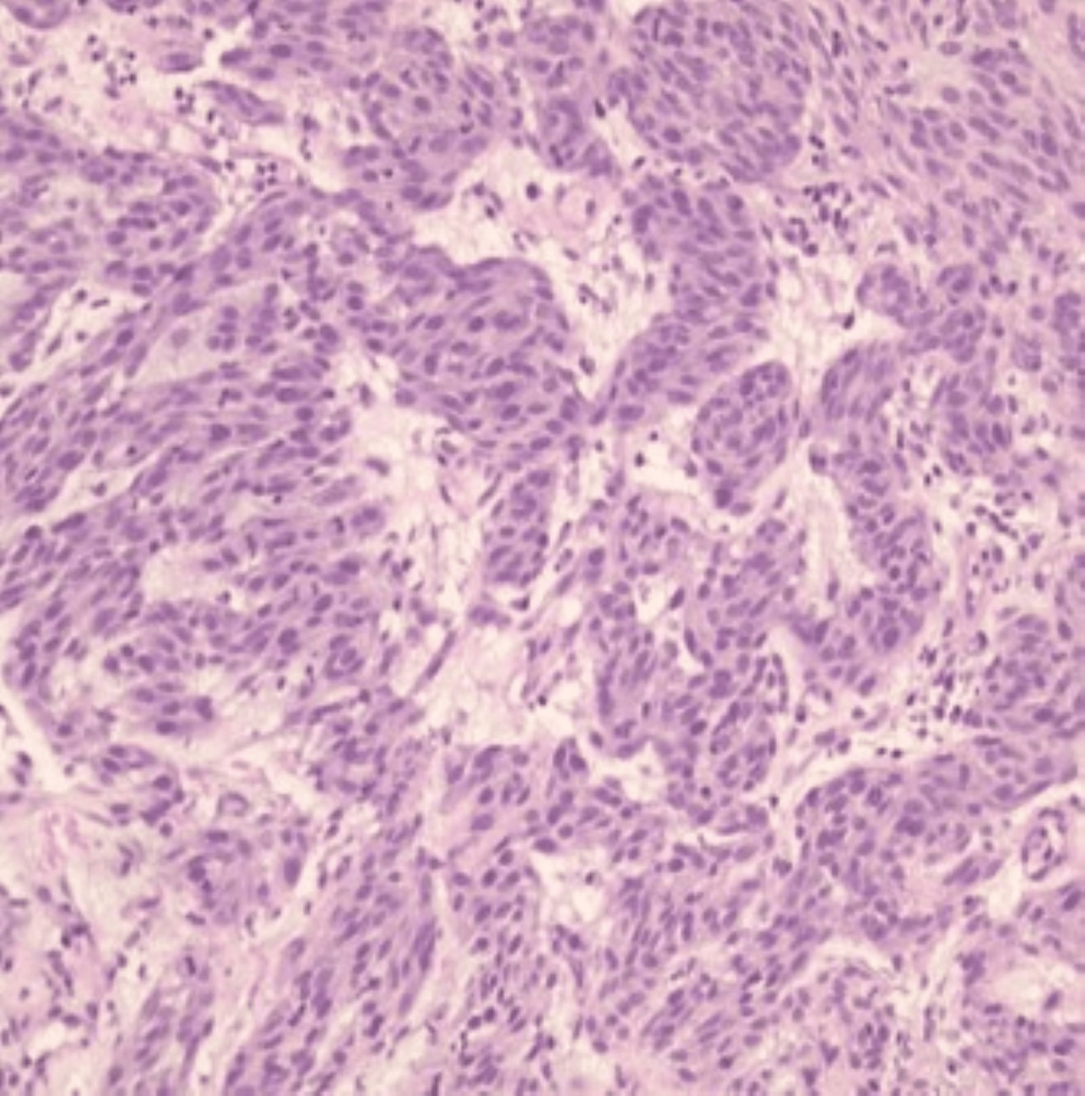
Gastric mucosa with underlying GIST in 10X view, showing spindle cells with eosinophilic cytoplasm GIST: gastrointestinal stromal tumor

**Figure 4:**
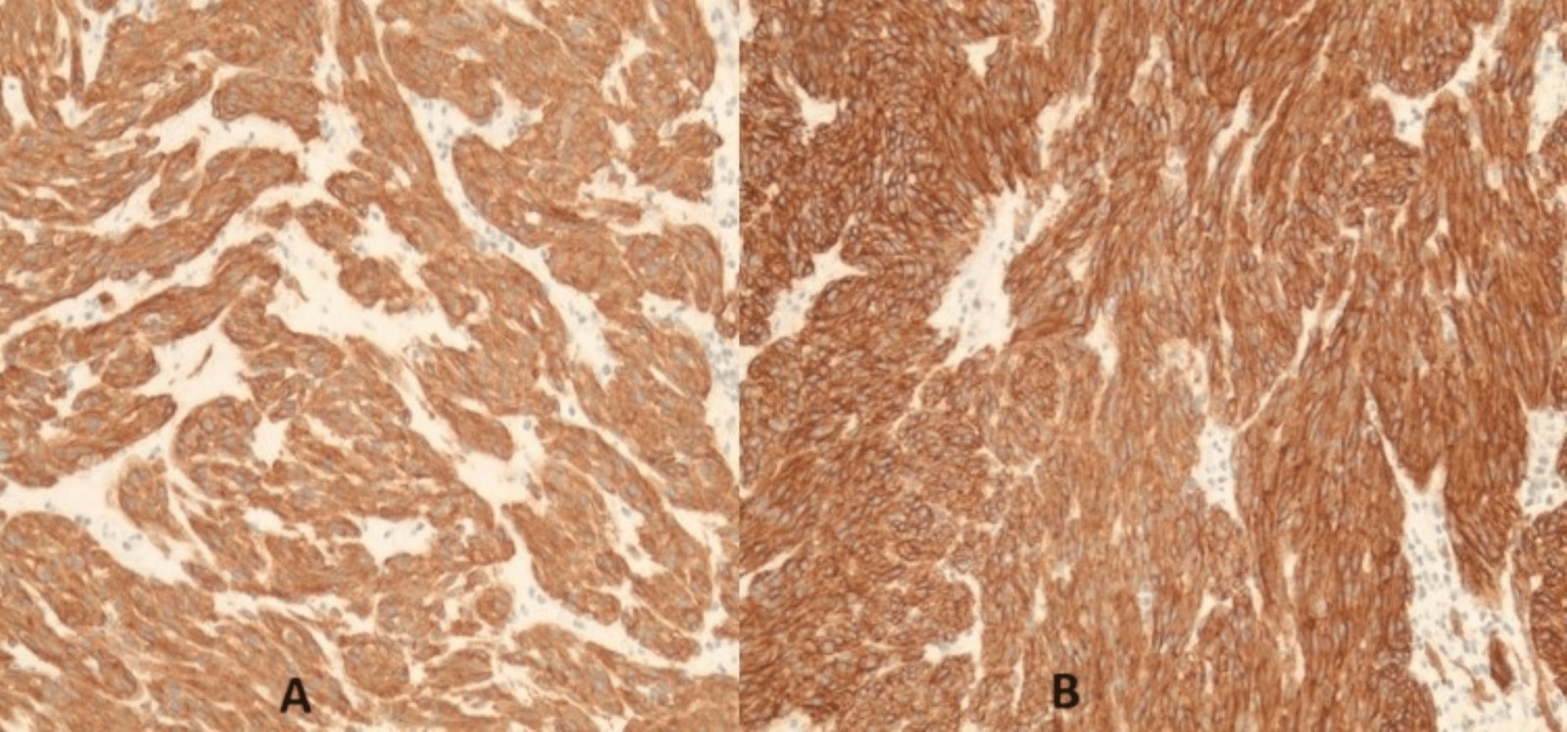
Immunohistochemical staining positive for c-kit stain (A) and DOG1 stain (B) c-kit or CD117 (cluster of differentiation 117) is a marker for hematopoietic stem cells. It is present in mast cell disease and gastrointestinal stromal tumors. DOG1 (discovered on gastrointestinal stromal tumors 1) is highly sensitive and specific for gastrointestinal stromal tumors.

A gastric cardia biopsy showed spindle cell type without necrosis, which was suggestive of high-grade GIST, as shown in Figure 3. The immunohistochemical study was positive for DOG1 (ANO1) and KIT (CD117), and negative for AE1/E3, as shown in Figure 4. Immunostaining for Helicobacter pylori was negative.

The tumor was staged to be a pT3N0 tumor. The patient was discharged on esomeprazole and iron supplements with a close follow-up with an oncologist and a surgeon. Hemoglobin was stable at 7.8 at the time of hospital discharge.

The patient had follow-ups with the oncologist and the surgeon. The patient underwent surgical resection with Roux-en-Y reconstruction after two weeks of hospital discharge, due to the risk of bleeding, obstruction, and perforation. The surgery revealed a 7 x 5x 4.5 cm tumor with negative margins. The patient was discharged from the outside facility, and the patient began follow-up with the oncologist outpatient. The pathology from the surgery revealed a high-grade malignancy, and the patient was started on empiric imatinib orally daily by the oncologist while awaiting the mutational study. The mutational study showed a KIT mutation of chromosome 4 (exon 11), and PDGFR alpha was negative. Since the patient had an exon 11 KIT mutation, the patient was continued on imatinib. The patient's hemoglobin stabilized above 9 after he received four iron sucrose transfusions, as he was a poor candidate for per oral iron supplement. The surveillance imaging showed interval partial gastrectomy with gastrojejunostomy without evidence of definitive malignancy or small bowel obstruction after three months of the imatinib therapy. The patient tolerated the treatment, has a positive response to the treatment, and has had a weight gain of 15 pounds since the surgery. The patient continues to follow up with the oncologist. Table 1 shows the timeline of the lab results.

**Table 1:** Lab results showing hemoglobin, MCV, platelet count, BUN, and creatinine values MCV: mean corpuscular volume; BUN: blood urea nitrogen

Date	Hemoglobin (g/dL)	MCV (fL)	Platelets (K/mcL)	BUN (mg/dL)	Creatinine (mg/dL)
01/04/2024	6	76.8	852	10	0.5
01/05/2024	8.1	79.8	648	7	0.5
01/09/2024	7.8	78.8	599	7	0.5
01/17/2024	7.3	79.3	573	8	0.4
4/4/2024	9	77.2	452	9	0.59
5/7/2024	11	77.6	466	9	0.59

## Discussion

We report a case of GIST in a young male who presented initially with abdominal pain, nausea, bloody vomiting, early satiety, black tarry stool, constipation, and weight loss. An upper endoscopy was done to investigate the etiology of upper GI bleed/black tarry stools, which revealed multiple esophageal and gastric ulcers. A biopsy with an immunohistochemical stain revealed the diagnosis of GIST. An extensive literature review showed that most of the reported cases had a median age of 60 years [7]. Less than 10% of the GIST is present in young adults less than 40 years of age [8]. Moreover, there are very few reported cases of GIST in the pediatric population with the median age reported at 13.5 years [9].

Immunohistochemical staining (CD117 and DOG1) is the most sensitive and specific diagnostic tool and yields a diagnosis in 98% of cases of GIST [10]. Our patient was positive for both CD117 and DOG1, and most importantly, the histopathology obtained by endoscopy revealed 0.3 cm spindle cells GIST of gastric cardia; hence, no genetic analysis was performed. The immunoreactivity for CD117 in GIST tumors is independent of mutational changes in KIT or PDGFRA gene, that is CD177 negative GIST can harbor KIT/PDGFRA mutations. These GISTs account for approximately 5% of the cases; thus, genetic analysis becomes imperative [11].

Although the mitotic activity and size are correlated to the malignant potential of tumors, the data suggests that even small GISTs with low mitotic activity can turn into malignant diseases [12]. For a non-metastatic and resectable GIST, surgery is the mainstay of the treatment. Our patient had a localized tumor of size >2 cm with high-risk features (irregular border, ulceration, and heterogeneity). Based on GEIS (Grupo Espanol Investigacion En Sarcomas) guidelines, surgical resection of the tumor was recommended in this patient [13]. Our patient had a successful surgical resection of the tumor and remained in remission on imatinib.

## Conclusions

GIST is a rare GI cancer, arising from stromal tissues in the GI tract, especially in the stomach and small intestine. The usual clinical presentations, especially in young patients with a negative family history of GI malignancy, mimic common GI pathologies like gastritis, and Zollinger-Ellison syndrome, and thus underscore the essentiality of correct diagnosis. 

A thorough history and proper investigations including imaging/endoscopy combined with immunohistochemical staining and genetic analysis should be pursued with a multidisciplinary approach as early detection is associated with a favorable prognosis. Treatment modalities for symptomatic GIST include medical or surgical management depending on various factors, including the tumor's size, resectability, potential for malignant disease, and extent of metastatic disease in case of metastatic tumors.
